# Does Race/Ethnicity Influence the Health of LGBT Older Adults? An Analysis of Adults Aged 50 and Older

**DOI:** 10.1093/geroni/igab046.2385

**Published:** 2021-12-17

**Authors:** Adrielle Magdael, Aditi Parashar, Aisha Ozair, Christi Nelson

**Affiliations:** 1 USF HPCC/USF School of Aging, TAMPA, Florida, United States; 2 University of South Florida, University of South Florida, Florida, United States; 3 University of South Florida, Tampa, Florida, United States

## Abstract

Lesbian, gay, bisexual, and transgender (LGBT) health disparities have been well documented in previous research; however, limited research has been conducted on racial/ethnic differences in health among LGBT older adults. Past research suggests that LGBT adults from racial/ethnic minority groups may encounter more discrimination and stigma than white LGBT adults, resulting in poorer health. This study investigated differences in general health between racial/ethnic groups in LGBT adults aged 50 and older from the 2018 and 2019 Behavioral Risk Factor Surveillance System annual surveys. The average ages were 64.2 years for the lesbian, gay, and bisexual (LGB) participants (n=3636) and 65.4 years for the transgender participants (n=972). For self-rated general health, the chi-square analysis indicated that there were significant differences between the racial/ethnic groups for both LGB and transgender participants, χ2(4, n=3630)=46.47, p<.001 and χ2(4, n=969)=19.03, p=.001, respectively. Logistic regression analyses found that, compared to White LGB adults, Hispanic LGB adults had higher odds (aOR=1.8, 95% CI=1.2-2.5) and Asian LGB adults had lower odds of reporting fair or poor health, (aOR=0.43, 95% CI=0.2-0.9). For transgender participants, Hispanic and Other Race adults had approximately twice the odds of reporting fair or poor health compared to White adults (aOR=2.1, 95% CI=1.2-3.7, and aOR=1.9, 95% CI=1.2-3.0, respectively).In conclusion, the results of this study suggest that cultural differences in racial/ethnic groups may influence the health of the LGBT community, making it an important factor to consider in research on LGBT older adults.

